# The Early Detection of Fraudulent COVID-19 Products From Twitter Chatter: Data Set and Baseline Approach Using Anomaly Detection

**DOI:** 10.2196/43694

**Published:** 2023-03-14

**Authors:** Abeed Sarker, Sahithi Lakamana, Ruqi Liao, Aamir Abbas, Yuan-Chi Yang, Mohammed Al-Garadi

**Affiliations:** 1 Department of Biomedical Informatics School of Medicine Emory University Atlanta, GA United States; 2 H Milton Stewart School of Industrial and Systems Engineering Georgia Institute of Technology Atlanta, GA United States; 3 Heinz College of Information Systems and Public Policy Carnegie Mellon University Pittsburgh, PA United States

**Keywords:** coronavirus, COVID-19 drug treatment, social media, infodemiology, public health surveillance, COVID-19, misinformation, natural language processing, neural network, data mining

## Abstract

**Background:**

Social media has served as a lucrative platform for spreading misinformation and for promoting fraudulent products for the treatment, testing, and prevention of COVID-19. This has resulted in the issuance of many warning letters by the US Food and Drug Administration (FDA). While social media continues to serve as the primary platform for the promotion of such fraudulent products, it also presents the opportunity to identify these products early by using effective social media mining methods.

**Objective:**

Our objectives were to (1) create a data set of fraudulent COVID-19 products that can be used for future research and (2) propose a method using data from Twitter for automatically detecting heavily promoted COVID-19 products early.

**Methods:**

We created a data set from FDA-issued warnings during the early months of the COVID-19 pandemic. We used natural language processing and time-series anomaly detection methods for automatically detecting fraudulent COVID-19 products early from Twitter. Our approach is based on the intuition that increases in the popularity of fraudulent products lead to corresponding anomalous increases in the volume of chatter regarding them. We compared the anomaly signal generation date for each product with the corresponding FDA letter issuance date. We also performed a brief manual analysis of chatter associated with 2 products to characterize their contents.

**Results:**

FDA warning issue dates ranged from March 6, 2020, to June 22, 2021, and 44 key phrases representing fraudulent products were included. From 577,872,350 posts made between February 19 and December 31, 2020, which are all publicly available, our unsupervised approach detected 34 out of 44 (77.3%) signals about fraudulent products earlier than the FDA letter issuance dates, and an additional 6 (13.6%) within a week following the corresponding FDA letters. Content analysis revealed *misinformation*, *information*, *political,* and *conspiracy theories* to be prominent topics.

**Conclusions:**

Our proposed method is simple, effective, easy to deploy, and does not require high-performance computing machinery unlike deep neural network–based methods. The method can be easily extended to other types of signal detection from social media data. The data set may be used for future research and the development of more advanced methods.

## Introduction

As of September 7, 2021, over 220 million confirmed COVID-19 cases have been reported globally, with over 41 million reported cases in the United States alone [1]. As governments and public health agencies worldwide made efforts to mitigate the impact of the pandemic, one persistent problem has been the opportunistic promotion of fraudulent products claiming to treat, prevent, test, or cure COVID-19 infections. The shortage of resources during the pandemic has allowed companies to exploit the public by selling them falsified products. These products include face masks, hand sanitizers, and test kits. Additionally, misinformation from social media has led to the usage of nonrecommended therapies such as ivermectin, methanol, and herbs and vitamins to prevent and treat COVID-19 infections [2]. Fraudulent products pose a threat to public health by inhibiting prevention and enabling the spread of disease, and by drawing people away from seeking recommended care. Furthermore, there have been numerous reports of adverse health events caused by toxic exposures to fraudulent products that have no scientific evidence supporting their use [3,4]. The Ministry of Health of Iran reported that between February and April 2020, there were 5011 patients with methanol poisoning and 505 confirmed deaths due to misinformation that methanol can neutralize COVID-19 [5].

In response to the emergence of many fraudulent products, the US Food and Drug Administration (FDA) has issued warning letters [6]. These warning letters are typically issued after the products become popular and many people have already been exposed to them. Between March and July 2020, approximately 3139 warning letters were released. Of those, 98 (3.14%) pertained to COVID-19–related products [7]. Since it is not possible to advertise fraudulent products on television or via reliable news sources, social media platforms have been exploited for the mass promotion of such products. In fact, promotional content regarding such products over social networks, such as Twitter, is only a subset of the misinformation spread through these platforms, which has been referred to as an *infodemic* [8,9]. The fraudulent products are often promoted directly via the social media accounts (eg, Twitter and Facebook) of the entities profiting from their sales, and, if the promotions gain traction, information about them are circulated by other social media users. It is estimated that from 2020 to 2021, there was a US $500 million consumer loss due to fraudulent products being sold [2]. Consequently, information regarding the products spread through social networks in analogous patterns as other types of misinformation, including those related to COVID-19 [10]. There is, thus, the need to develop toxicovigilance tools that can automatically identify potentially fraudulent COVID-19 products early and generate alerts. While social networks provide fertile grounds for the proliferation of misinformation about fraudulent products, they also provide opportunities for responding to diverse challenges posed by the pandemic, and one potential utility of social media is the automated real-time surveillance of fraudulent COVID-19 products.

In this paper, we demonstrate that chatter about fraudulent products on Twitter, if curated systematically via natural language processing (NLP) and data-centric methods, can provide detectable early signals. We used publicly available streaming data from the Twitter COVID-19 application programming interface (API), which was specifically created by the company to aid COVID-19–related research [11]. Specifically, using Twitter data, we show that social media–based surveillance can detect many fraudulent products early, relative to the FDA warning issuance dates. Our approach to detecting fraudulent products is based on a simple intuition—that products that gain popularity among Twitter users, following their successful promotion, will exhibit increases in their mentions in COVID-19–related chatter. These abrupt increases in the frequency of mentions are likely to be detectable through time-series anomaly detection methods. It is also likely that products that gain *relatively* higher popularity will exhibit anomalous increases of *relatively* higher magnitudes in their mentions among all COVID-19–related Twitter chatter. We present our findings in the following section and detail our methods at the end of the article.

## Methods

### Ethical Considerations

This study was reviewed by Emory University’s institutional review board, which determined on June 11, 2020, that it was exempt from further review (category 4), since only publicly available data were included (STUDY00000711).

### Data Collection

We collected data using the COVID-19 streaming API of Twitter [11]. This API was made available by Twitter specifically for supporting COVID-19–related research, and it does not impose throughput limitations or daily or monthly quotas. Consequently, we were able to collect all tweets that mentioned COVID-19–related keywords and phrases (eg, *coronavirus*, *covid19*, and *covid*) [11]. We collected data from February 19 to December 31, 2020. Streaming data were stored in real time in a *mongodb* database hosted on the Google Cloud platform. The collection of data was continuous with only minor down times that were necessary for system modifications or updates.

### Product Detection

The list of products and entities were manually collected from the FDA website [6]. The products included were advertised as treatments or cures, tests, or preventative measures for COVID-19. We curated a comprehensive list of entity names, products, FDA letter dates, persons who owned the entities or the products, websites, and social media profiles (if any). We curated this information for a total of 183 letters issued by the FDA. Each warning letter was manually reviewed. From these, we manually curated a set of product names or entity names that were potentially used for promotion over social media. If the same product was mentioned in multiple letters, we only included the first mention of the product or entity and the corresponding date, excluding the later ones. We also manually curated keywords and phrases that were likely to be used to refer to the products or entities on Twitter. The full list of products and entities and their earliest letter dates is provided in Table 1.

Since product and entity names are often misspelled by social media subscribers, keyword-based searches typically miss large numbers of posts that contain misspelled versions of the names. To increase the sensitivity of our searches, we applied NLP to increase the number of keywords we searched for that were associated with each product or entity. Specifically, we generated potential spelling variants or misspellings of the products and entities using a previously developed data-centric tool [12]. The variant generation tool uses a combination of semantic and lexical similarity measures to automatically identify common misspellings and spelling variants of terms or phrases, including multiword expressions. Our past work revealed that such lexical expansion strategies are capable of significantly increasing retrieval or detection rates from Twitter, particularly for medical terms (eg, names of medications) that are often difficult to spell [13]. Examples of product names extracted from the warning letters and their automatically generated lexical variants are shown in Table 2. We included all products or entities and their spelling variants that had at least 10 mentions in our collected data. We excluded key phrases that were mentioned less than 10 times because such low occurrences indicated that the corresponding products or entities were either not promoted over Twitter or never actually gained popularity on the platform. We enumerated the mentions of each product or entity, including their spelling variants, from the entire collected data set. Counts of spelling variants were grouped with the original products or entities. Daily counts were normalized by the total number of posts collected on the same days. The daily relative frequencies were represented as the number of mentions per 1000 tweets.

**Table 1:** Key phrases included in this study along with their types and the date of the first letter mentioning each of them.

Number	Key phrase	Type	First detected letter date
1	Antimicrobial solution	Treatment	November 2, 2020
2	Aromatherapy	Treatment	March 6, 2020
3	Ayurvedic products	Treatment	April 13, 2020
4	Bee products	Treatment	October 23, 2020
5	Berberine	Treatment	October 23, 2020
6	Betterfly	Treatment	September 1, 2020
7	Bioflavonoids	Treatment	October 23, 2020
8	Biomagnetism	Treatment	August 19, 2020
9	Chlorine dioxide	Treatment	April 8, 2020
10	Cod liver oil	Treatment	May 25, 2020
11	Colloidal silver	Treatment	March 6, 2020
12	Colostrum	Treatment	May 26, 2020
13	Corona-cure	Treatment	March 26, 2020
14	Covid-19 rapid test kit	Test kit	June 10, 2020
15	Curativa	Treatment	June 25, 2020
16	Elderberry syrup	Treatment	November 10, 2020
17	Elderberry tincture	Treatment	March 6, 2020
18	Essential oil	Treatment	March 6, 2020
19	Eupatorium perfoliatum	Treatment	March 6, 2020
20	Grapefruit seed extract	Treatment	May 26, 2020
21	Hypochlorous acid	Treatment	November 2, 2020
22	Iodine products	Treatment	June 10, 2020
23	Kratom	Treatment	May 15, 2020
24	Magnetic therapy	Treatment	August 19, 2020
25	Methylene blue	Treatment	May 29, 2020
26	Nad+	Treatment	May 6, 2020
27	Nephron pharmaceuticals	Treatment	May 22, 2020
28	Niacin product	Treatment	September 1, 2020
29	Novabay	Entity	November 2, 2020
30	Oracare	Treatment	November 18, 2020
31	Pro breath	Treatment	November 18, 2020
32	Quercetin	Treatment	June 15, 2020
33	Salt therapy	Treatment	March 30, 2020
34	Santiste	Entity	April 27, 2020
35	Super C	Treatment	April 21, 2020
36	Superblue silver immune gargle	Treatment	April 9, 2020
37	Supersilver whitening toothpaste	Treatment	April 9, 2020
38	Traditional Chinese medicine	Treatment	May 8, 2020
39	Transdermal patch/defendTM patch	Treatment	April 27, 2020
40	Umbilical cord blood	Treatment	June 4, 2020
41	Vapore	Treatment	July 30, 2020
42	Vidacord	Treatment	June 4, 2020
43	Vivify	Entity	March 6, 2020
44	Xosomes	Treatment	June 4, 2020

**Table 2:** Examples of fraudulent product names extracted from the US Food and Drug Administration’s warning letters and their automatically generated lexical variants.

Product	Spelling variants
Chlorine dioxide	*chlorinedioxide*, *chloride dioxide*, *chorine dioxide*, *clorine dioxide*, and *clorinedioxide*
Fortify humic beverage concentrate	*fortify humic beverage concentrates* and *fortify humic beverage cocentrate*
Electrify fulvic beverage concentrate	*electrify fulvic beverage cocentrate*, *electrify fulvic beverage concetrate*, and *electrify fulvic beverage concentrates*
Supersilver whitening toothpaste	*supersilver whitening toothpast*, *supersilver whitening toothpastes*, and *supersilver whitening tooth paste*
Superblue fluoride free toothpaste	*superblue fluoride free tooth paste*, *superblue fluoride free toothpastes*, and *superblue fluoride free toothpast*
Prefense hand sanitizers	*prefense handsanitzers*, *prefense hand sanitizes*, *prefense hand sanitiers*, *prefense hand andsanitizers*, *prefense hand*, *prefense hand handsantizers*, *prefense hand handsanitzers*, *prefense handsantizer*, *prefense handsanitizers*, *prefense*, *prefense hand santitizers*, *prefense handsanitisers*, and *prefense handsanitzer*
Covid-19 cough syrup	*covid 19 cough syrups*, *covid 19 coughsyrup*, *covid 19 cough syrup*, and *covid 19 cough coughsyrup*
nCov19 spike protein	*ncov19 spike spike protein*, *ncov19 spike spikeproteins*, *ncov19 spike protei*, *ncov19 spikey proteins*, *ncov19 spike spikeprotein*, *ncov19 spikeprotien*, *ncov19 spike proteins*, *ncov19 spike spikey proteins*, *ncov19 spikeprotein*, *ncov19 spikeproteins*, and *ncov19 spike spikeprotien*

### Detecting Anomalies

We applied a 14-day moving average filter to construct a smooth line representing the daily mention frequencies, and anomalies or outliers were detected relative to this moving average line. For each day, the *residual* for SD calculation was computed by subtracting the 14-day moving average from the relative frequency per 1000 tweets on that day. For a given day (*n*), the SD for the day (*σ_n_*), is computed progressively, given as follows:




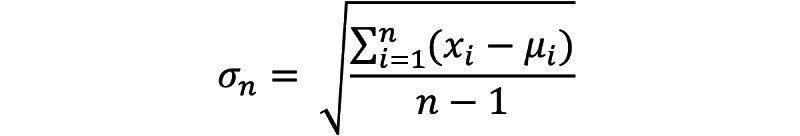




where *x_i_* is the relative frequency for day *i* and *μ_i_* is the 14-day moving average on day *i*. Thus, the SD computed for a given day includes all the data points starting from day 1. The SD for the first day (February 19, 2020) for any product is by definition 0. This may potentially give the anomaly detection approach an unfair advantage by increasing the sensitivity of detection in the early days easier. Therefore, we artificially added a nonzero SD on day 1, computed as:




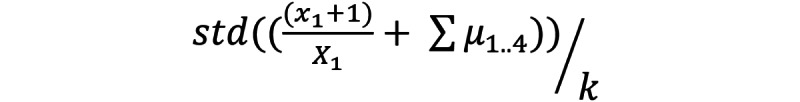




where *x_1_* is the product mention frequency on day 1, *X_1_* is the total number of tweets collected on day 1, *std*() is the SD function, and *μ*_1..4_, are the moving averages over the first 4 days. The value is divided by *k* to adjust for the ***k*** × *std*()function that is applied to compute the boundaries beyond which a data point would be considered an outlier (***k***=3 in our experiments). This artificial initial bias that we added, therefore, decreases the chances of our approach to detect outliers early on in the time lines and makes the task of detecting anomalies slightly more difficult, particularly for products that have a relatively low number of mentions. For some products, for example, there are many days with 0 mentions early on in their time lines, but the added bias causes the progressive SD to be nonzero. For 3 products with letter issue dates in March 2020, this added bias caused the method to miss early outliers that are detectable without adding the bias. Specific details are provided in Multimedia Appendix 1. The minimum value for daily relative frequency was set at 0.001 (ie, ***k*** × 0.001 served as the minimum threshold for outlier detection).

The chosen window size (14) and SD (3), for which we report results in this paper, were relatively conservative choices for signal detection. We also performed experiments with multiple window sizes (7, 10, and 14) and SD thresholds (2, 2.5, and 3) to study how the anomaly detection performance varied on the basis of these parameters. Slight variations in window sizes and SD did not impact overall performance.

### Evaluation

Data points that had a distance of more than 3 SDs from the moving average were considered outliers (ie, signals). For each key phrase, the date of the first outlier was compared with the FDA letter issuance date to determine if the signal was detected earlier, within 1 week, or later than the FDA letter issuance date. System percentage accuracy was computed using the formula: #*early*/#*total*. For products that were mentioned in multiple letters, our approach was only considered successful in early detection if the outlier was detected prior to the first mention date. Thus, the reported system performance is actually likely to be lower than that in practice.

### Content Analysis

To obtain an idea about the contents of the Twitter posts associated with the fraudulent products, we performed a brief, manual content analysis of 400 posts associated with 2 products (200 each). The 2 products chosen—*chlorine dioxide* and *quercetin*—had over 10,000 posts in the data set each and were among the top 5 most frequently mentioned. We performed random sampling to select the posts for manual review. Two authors manually reviewed the posts and identified possible categories for the posts. Following the first round of coding, the categories were collapsed into broader topics. Finally, we computed the distributions of these topics among the manually categorized posts.

## Results

The issue dates of the letters ranged from March 6, 2020, to June 22, 2021. Through manual review of each letter, we identified 221 potential keywords or phrases that were either associated with the products (eg, product names) or the entities selling them. From this set, we excluded key phrases collected after the year 2020. Some products were promoted by different entities at different times, causing them to be repeated in the warning letters. Since our primary objective was to assess the possibility of early detection, we excluded repeated key phrases, retaining only their first occurrences (n=56). Furthermore, since our focus was to detect products that gained popularity via promotion on Twitter, we excluded key phrases that were mentioned less than 10 times, including their lexical variants (n=12). In total, 44 key phrases met all the inclusion criteria. Table 1 presents all 44 keywords, their types (ie, product or entity), and the FDA letter issuance dates. The full curated data along with additional information is available in Multimedia Appendix 2.

**Figure 1:**
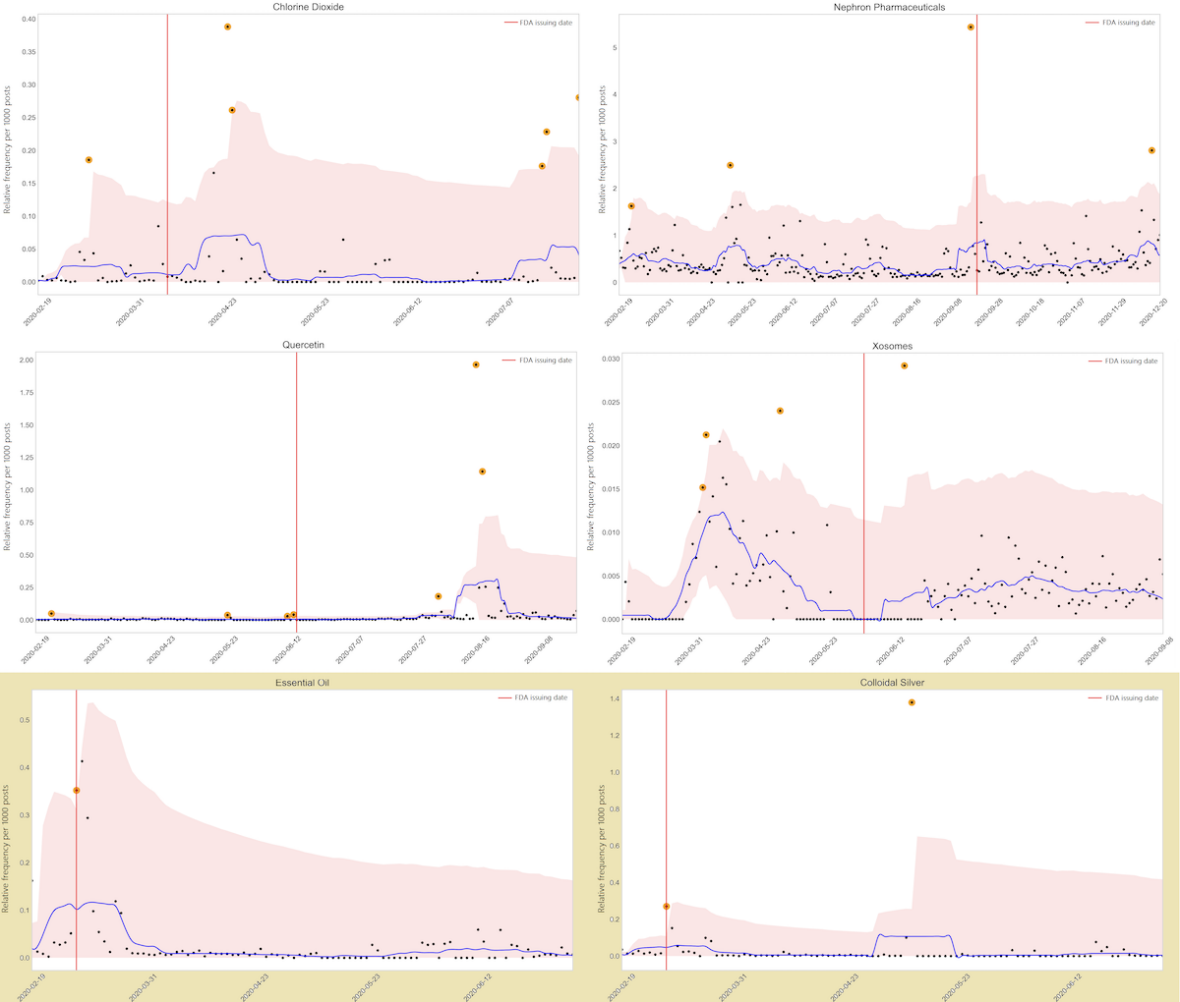
Daily relative frequencies for 6 sample products or entities from our data set, their 3-SD ranges, and the moving averages. FDA: US Food and Drug Administration.

We included a total of 577,872,350 COVID-19–related tweets in our analysis, which were collected from February 19 to December 31, 2020. We computed the daily counts of the key phrases (along with their spelling variants, if any). Increases in the number of key phrase mentions that were higher than 3 SDs from the 14-day moving average of mentions were flagged as potential “signals.” In total, 43 out of the 44 key phrases showed anomalous increases in their mentions at some point of time within our collected data. For 34 out of the 44 (77.3%) key phrases, signals of anomalous increases in chatter were detectable prior to the FDA letter issuance dates. An additional 6 (13.6%) key phrases had anomalous increases within 7 days of the FDA letter issuance dates. Figure 1 presents the daily relative frequencies for 6 sample products or entities from our data set, their 3-SD ranges, and the moving averages. The top 4 panels in the figure represent products or entities for which anomalies were detected prior to the FDA letter issue dates and the bottom 2 panels (highlighted in beige in Figure 1) represent those for which anomalies were not detected prior to the letter issue dates. A larger figure with all 44 products or entities are provided in Multimedia Appendix 3. The daily counts for all 44 key phrases are provided in tabular format in Multimedia Appendix 4.

Table 3 presents the distribution of the topics in terms of percentage for the 2 products identified via manual analysis. We discovered 4 prominent topics—*misinformation*, *information*, *conspiracy theories*, and *political.* Posts that could not be categorized as any of these were labeled as *other.* Misinformation included the spreading of information that these products cure or treat COVID-19. They also consisted of marketing and promotion of these products. Some posts claimed that the user took these products to successfully recover from COVID-19. Particularly for quercetin, many posts encouraged the consumption of multiple dietary supplements such as zinc and vitamin C alongside quercetin. Some of the posts also shared unverified news articles that claimed high efficacy of these products against COVID-19. Many posts shared reliable information and news that countered the unverified claims. Posts belonging to the *information* category also mentioned the FDA letters that we discussed in this paper. We also came across a number of posts that were spreading conspiracy theories, which included false claims about the vaccine or suggestions that the government was intentionally suppressing information about the efficacy of these products. Posts that were categorized as *political* included those that tagged politicians, commented on statements made by politicians, or discussed political mandates. Note that while the proportion of misinformation appears higher for quercetin, many posts that mentioned it were simply speculations about its effectiveness in preventing COVID-19, and the posts often referred to or recommended other forms of protection as well, such as masking. For consistency, we grouped such speculations and advice as misinformation.

**Table 3:** Distribution of topics in the manually reviewed posts about chlorine dioxide and quercetin.

Topics	Chlorine dioxide, %	Quercetin, %
Misinformation, marketing, or promotion	37.0	58.0
Information or news	32.5	30.5
Conspiracy theories	13.5	4.0
Political	7.0	2.5
Other	10.0	4.0

## Discussion

### Principal Results

The primary finding of this study is that our proposed approach allows for anomaly detection in Twitter chatter that is typically associated with a fraudulent product or entity. This method, combined with further in-depth content analysis, can potentially enable us to detect fraudulent products early—as they start getting popular—from Twitter. Since social media serves as a platform for promoting such fraudulent products, increases in their popularity are also likely to cause increases in their web-based mentions. This phenomenon potentially makes it possible to detect fraudulent products that are rising in popularity to the point that renders them a public health concern. Thus, while social media plays an important role in the spread of information about fraudulent products, and misinformation in general, it may also serve as a potential resource for the surveillance of such information. While other information sources are often laggy, social media provides the opportunity to conduct surveillance in close to real time. While our approach is relatively simple, it is very effective in detecting fraudulent products that rise in popularity. Determining the contents of the chatter and the specific dangers that may arise from the content requires further analysis, which is beyond the scope of this study, and we intend for such analyses to be carried out in future work. There is also the potential of developing more advanced and effective methods for detecting such fraudulent products.

In addition to our approach, the data set curated from publicly available FDA reports can help drive future research in this space. To the best of our knowledge, there is no such data set that has been curated and is available for research. Thus, the data set itself can be of high utility to the research community. Importantly, the data set can serve as an important resource for the development of methods to detect misinformation in general from social media data.

### Limitations

There are several potential limitations of the proposed approach. First, it requires data that are not rate-limited (eg, data from the standard Twitter streaming API). Anomalous increases may not be detectable from rate-limited streams, since large increases in volume are likely to be dampened by the APIs. For real-time detection of fraudulent product candidates, deployment needs to be performed on streaming data, although it is also possible to periodically run the anomaly detection scripts on stored, static data. Second, we were only able to calculate the percentage of early detection within our given sample, and based on the current data, we were unable to realistically estimate CIs for the percentage values reported. Third, the anomaly detection approach relies on characteristic abrupt increases in chatter volumes about a given topic. It is possible that some fraudulent products may gain popularity gradually, causing the normalized counts to never exceed the SD threshold. In such cases, varying the window size (eg, using 7-day moving averages) and lowering the SD thresholds may improve the detection capability of the method. However, lowering the SD threshold is also likely to result in larger numbers of false positives—an aspect that we did not take into account in this study. We believe that not taking false positives into account in this study is justifiable, since in practical settings, all signals associated with noun phrases would be reviewed by experts; hence, it is perhaps better if the method is biased in favor of *recall* (ie, more true and false positives) rather than *precision.*

We also do not address the detection of candidate fraudulent substances in this study. Several mechanisms can be used for detecting candidates including, but not limited to, named entity recognition (likely to be high precision but low recall), simple part-of-speech tagging to identify noun phrases (high recall and low precision), and topic modeling methods that identify possible topics from texts (low recall and high precision). We intend to explore these strategies in future work. Even without this component, we believe our approach is an improvement over past studies that did not take into account the warning letter dates. We also did not conduct in-depth analysis of the content associated with all the included products or entities or the features associated with the accounts that post the information. Both of these are important future research directions. Finally, since the daily counts are normalized by the total number of tweets on the same day, it is possible that large increases in absolute counts of specific key phrases are not detectable due to equal or larger increases in the total volume of posts on the same day.

### Comparison With Prior Work

Our work is not the first to explore the utility of social media as a potential source for detecting fraudulent COVID-19 products. In recent studies, unsupervised NLP methods such as topic modeling and supervised methods such as text classification have been proposed for the automatic detection of such products from social media data [14-16]. Others focused more broadly on detecting misinformation using social media or internet-based data [17,18]. However, these studies did not take into account the time factor. Typically, once the FDA issues a warning about a fraudulent product, there is a rise in chatter regarding the product, but such rises are driven by media coverage or increased public awareness. We observed this phenomenon for most products included in the study, particularly the ones detected within 1 week of the FDA letter issuance dates. Some recent studies have conducted more in-depth analyses of misinformation associated with specific products or substances that were rumored to be effective against COVID-19. For example, Kim et al [19] fine-tuned transformer-based models to automatically classify misinformation related to garlic. Quinn et al [20] analyzed misinformation related to vitamin D and COVID-19 on YouTube. A larger set of studies has focused on COVID-19–related misinformation on social media, in general, for topics such as, for example, vaccines [21-23]. To the best of our knowledge, our approach is the first to attempt to detect fraudulent treatments early. The proposed approach is also simple and computationally inexpensive as it relies on fundamental characteristics of social media chatter (ie, increases in the volume of chatter about a particular topic resulting from increases in its popularity) and is unsupervised (ie, no training data required).

### Conclusions

The emergence of fraudulent products associated with COVID-19 has been a significant problem in the fight against the pandemic. Social media has served as a platform for advertising and promoting fraudulent products. While social media makes it easier for opportunist entities to promote and sell fraudulent products, this resource may also be used to conduct surveillance of fraudulent substances. In this paper, we show that it is possible to detect many fraudulent products potentially early from Twitter data. Our simple approach used a time-series anomaly detection method for detecting anomalous increases in mentions of fraudulent substances in Twitter chatter and obtained promising performance. Future work will focus on deploying the NLP pipeline and improving upon the study limitations.

## Appendix

Multimedia Appendix 1 Additional system performance details.

Multimedia Appendix 2 Full details about the fraudulent products.

Multimedia Appendix 3 Daily counts for all 44 products/entities from our dataset, their 3-standard deviation ranges, and the moving averages.

Multimedia Appendix 4 Daily counts for all included key phrases.

## Abbreviations

API: application programming interface
FDA: US Food and Drug Administration
NLP: natural language processing
