# Supplementary material for: The Early Detection of Fraudulent COVID-19 Products From Twitter Chatter: Data Set and Baseline Approach Using Anomaly Detection
Source: JMIR Infodemiology. 2023 Mar 14;3:e43694. doi: 10.2196/43694 (PMC10131818; doi:10.2196/43694)
Supplement: Multimedia Appendix 1 [file infodemiology_v3i1e43694_app1.docx]

**SUPPLEMENTARY MATERIAL (MULTIMEDIA APPENDIX 1)**

**Additional performance details**

Additional products that were detectable if the initial bias was not added:

*Colloidal silver* (FDA letter date: 03/06/2020)

*Essential oil* (FDA letter date: 03/06/2020)

*Super C* (FDA letter date: 04/21/2020)
